# Perceived challenges in treatment decision-making for endometriosis: healthcare professional perspectives

**DOI:** 10.1080/21642850.2024.2383469

**Published:** 2024-08-01

**Authors:** Lynda Fallon, Annie Y.S. Lau, Donna Ciccia, Tanya Jane Duckworth, Chantelle Pereira, Emily Kopp, Valentina Perica, Kerry A. Sherman

**Affiliations:** a School of Psychological Sciences, Macquarie University, Sydney, Australia; b Lifespan Health and Wellbeing Research Centre, Sydney, Australia; c Australian Institute of Health Innovation, Macquarie University, Sydney, Australia; d National Institute of Complementary Medicine (NICM), Health Research Institute, Western Sydney University, Sydney, Australia; e Endometriosis Australia, Sydney, Australia; f School of Biomedicine, Faculty of Health and Medical Sciences, University of Adelaide, Adelaide, Australia; g School of Health Sciences, Faculty of Medicine and Health, University of Sydney, Sydney, Australia

**Keywords:** Endometriosis, shared decision-making, treatment, qualitative, healthcare professionals

## Abstract

**Background:**

Endometriosis, a systemic chronic inflammatory condition which has no cure, has a high symptom burden that can negatively impact every facet of life. Given the absence of a gold-standard treatment, the best symptom management regimen in endometriosis is heavily reliant on a patient's values and preferences, making shared decision-making (SDM) vital. However, a comprehensive patient decision aid (PtDA) intervention that could facilitate patient decision-making and promote SDM is lacking in endometriosis, and there is little research on the decisional support needs of individuals with this condition. This qualitative study aimed to explore healthcare professional (HP) perspectives of their clients’ decisional support needs when choosing treatments to manage endometriosis symptoms, with a view to evaluating the need for a PtDA.

**Methods:**

Australian HPs identified as specialising in endometriosis care (*N* = 13) were invited to participate in a short interview over the Internet by phone. Questions focussed on perceived facilitators and challenges of decision-making when choosing treatments for endometriosis. Transcribed qualitative data were thematically analysed and verified by multiple coders, using the template approach.

**Results:**

Four themes were identified: (1) Identifying and setting priorities; (2) HPs’ lack of time and perceived lack of knowledge; (3) Patient-centred care and SDM, including patient capacity; and (4) Decision-making blinded by hope. This is the first known study to explore HPs’ perspectives on patient decision-making challenges in endometriosis.

**Discussion:**

Findings draw attention to the difficulties people with endometriosis experience when assessing and choosing treatments, highlighting the need for a comprehensive PtDA intervention to support this decision-making.

Endometriosis is a chronic inflammatory disease affecting one in seven biological women of reproductive age in Australia (Australian Institute of Health and Welfare [AIHW], 2023; Rowlands et al., 2021). Symptoms, including severe and chronic pelvic pain, dysmenorrhea and dyspareunia, can be debilitating (Sivajohan et al., 2022), negatively impacting quality of life, social and personal relationships, work, education and sexual functioning (Calvi et al., 2024; Chapron et al., 2019; Sullivan-Myers et al., 2023). Psychosocial impacts of endometriosis include heightened levels of fatigue, stress, anxiety and depression (Sullivan-Myers et al., 2023; Gambadauro et al., 2019; Pope et al., 2015).

There is no cure for endometriosis. Treatments aiming to manage symptoms (O’Hara et al., 2022) include medications (e.g. NSAIDs, hormonal), surgery to remove endometriotic lesions or affected organs (e.g. hysterectomy) (Chapron et al., 2019), and psychotherapies, and allied health and complementary therapies (D’Alterio et al., 2022). However, there is no gold-standard treatment; all options offer differing advantages and disadvantages, and varying effectiveness, depending on the individual (Colón-Caraballo et al., 2019; Nirgianakis et al., 2021). Consequently, optimal endometriosis treatment amalgamates clinical characteristics and the patient's informed preferences regarding each option (Geukens et al., 2018; Llewellyn-Thomas & Crump, 2013).

The lack of a single definitive treatment plan for endometriosis makes it an ideal candidate for shared decision-making (SDM), the preferred approach for health-related decision-making when multiple legitimate options exist (Stacey et al., 2020), and in chronic conditions where decision-making is ongoing (Wieringa et al., 2019). Although studies report that SDM leads to optimal choices that are more likely to be actioned (Barry & Edgman-Levitan, 2012), across a range of healthcare contexts clinicians frequently do not use SDM (Stalnikowicz & Brezis, 2020), and consumers do not feel empowered to participate in the process (Joseph-Williams et al., 2014; Keij et al., 2021). Furthermore, people living with endometriosis (PLWE) report challenges in communicating with healthcare professionals (HPs) (Sherman et al., 2022), and feel dissatisfied with how their symptoms are managed (Evans et al., 2022), alone in making treatment decisions, and overwhelmed when trying to navigate options (Handelsman et al., 2023; Metzemaekers et al., 2021). This suggests they could benefit from support in making these important and complex decisions (Geukens et al., 2018; Vercellini et al., 2018). A robust evidence base across different health screening or treatment contexts demonstrates the effectiveness of patient decision aids (PtDAs) in enhancing patient knowledge about their condition and providing clarity on their personal values (Stacey et al., 2017). Importantly, PtDAs enhance communication between PLWE and clinicians, facilitating SDM (Wieringa et al., 2019).

Best practice for creating a PtDA requires a codesign process with input from both sides of SDM: PLWE and HPs (Coulter et al., 2013; Witteman et al., 2021a, 2021b). However, clinicians’ perspectives on the support required by PLWE when deciding on endometriosis treatments have not been canvassed. This study aimed to address that gap by qualitatively exploring HP perspectives on the challenges faced by their PLWE clients when making decisions about endometriosis treatments, with a view to determining whether a PtDA could be helpful. Due to the exploratory nature of this study, semi-structured interviews were deemed the best data collection method (Adeoye-Olatunde & Olenik, 2021; Busetto et al., 2020).

This study aimed to ascertain HP perspectives on: (1) Whether PLWE feel they can fully participate in SDM when choosing treatments for their endometriosis; (2) Which aspects of endometriosis treatment decision-making PLWE find most difficult; and (3) Whether a PtDA could support and facilitate the decision-making process.

## Materials and methods

### Participants

In an attempt to gather a diversity of viewpoints, Australian-based HPs specialising in endometriosis treatments and management, including medical, surgical, psychological, and complementary and allied health options, were identified through: Australian Government specialist pelvic pain clinics (Australian Government [Internet], 2023); endometriosis consumer support organisations (i.e. Endometriosis Australia, EndoZone); and the research team's networks. An email was sent to 63 HPs inviting them to a 15- to 20-minute phone or Zoom interview. Sixteen HPs agreed to participate, but three could not find a suitable time, leaving *N* = 13: 3 obstetrician-gynaecologists, 5 specialist clinic staffers (3 general practitioners (GPs), 1 practice manager, 1 practice nurse), 2 allied HPs (psychologist, physiotherapist), and 3 complementary medicine HPs (dietitian, naturopath and wellness coach), representing major healthcare options for endometriosis symptom management (Becker et al., 2022; Kalaitzopoulos et al., 2021). The HPs had specialised in endometriosis care for between 4 and 30 years (*M* = 14.5 years). The sample size was deemed sufficient to explore the topic (Hennink et al., 2019), which was confirmed when no new codes were raised from interview 11 onwards (Silverman, 2014).

### Procedure

Two telephone and 11 Zoom interviews were conducted between May and October, 2023, with each lasting 14-49 min, depending on the HP. Participants provided verbal consent to record the interview with LF (a middle-aged female student trained in psychology), and were all asked the same set of iteratively designed open-ended questions (Supplementary Materials A) about their perceptions of treatment decision-making by their PLWE clients. Prior to any interviews, the question set was piloted by the research team, including two consumer investigators (DC and TD), who are trained researchers with lived experience of endometriosis and who are affiliated with peak consumer body Endometriosis Australia. The study was approved by an institutional human research ethics committee (Reference no: 520231301346445).

### Data analysis

Recorded interviews were uploaded to Microsoft Word's Transcribe function for transcription, and the accuracy of the transcripts was checked against the recordings by LF, EK and VP. Transcribed data were de-identified before analysis, with each participant referred to by their profession. No HP took up the offer of checking the transcript of their interview.

The template approach was used to assess these data, because this inductive method of thematic analysis enables a flexibility which is ideally suited to exploratory research (Brooks et al., 2015). The steps were (1) Dataset familiarisation: LF and CP read the transcripts. (2) Generation of initial codes: LF and CP independently identified meaningful clusters of data to derive initial codes from the transcripts of the first six interviews. (3) Initial theme formation: Following comparison and mutual agreement on preliminary themes, LF and CP proceeded to code the remaining interviews. (4) LF examined the commonalities and distinctions among coded items, consolidating them before seeking feedback from CP. KS oversaw this process to ensure uniformity across codes and coders. (5) LF and CP iteratively consolidated the themes, resulting in a final coding template of four themes with two subthemes. At this stage the focus of each theme was honed, labels were assigned, and the researchers deliberated about how each addressed the research questions. (6) The written report used the themes to communicate the findings, accompanied by pertinent quotes from participants.

This research is reported according to COREQ guidelines (Tong et al., 2007). A critical realist ontological perspective guided this study (Fletcher, 2017), coupled with an interpretive approach (Olmos-Vega et al., 2023). Due to the sensitivity of the data, it is not publicly available.

## Results

Four themes were identified from these data analyses: Identifying and Setting Priorities (sub-theme: Stepwise Approach to Treatment); HPs’ Lack of Time and Perceived Lack of Knowledge; Patient-Centred Care and SDM (sub-theme: The Effect of Patient Capacity on Decision-Making); and Patient Decision-Making Blinded by Hope. Salient HP quotes illustrate each theme and sub-theme. In recognition of the wide range of treatments used to manage endometriosis symptoms (Leonardi et al., 2020), and of the key role practice managers/nurses and non-medical clinicians can play in helping to guide PLWE (Norton & Holloway, 2020), effort was made to include HPs from disparate health fields to canvas as wide a range of viewpoints as possible. However, there was remarkable consistency in participants’ responses, reflecting the unanimous views of this sample to strive for patient-centred care and the need for a multi-disciplinary approach to endometriosis symptom management. Although the focus of this study was HP perspectives on treatment decision-making in PLWE, interviews frequently organically included discussions about how participants approached care provision. These insights enriched understanding of possible ways to support the decision-making process for PLWE, and thus have been included. Additional quotes are provided in Supplementary Materials B.

### Theme 1: identifying and setting priorities

HPs were almost unanimous about the need for PLWE to prioritise which symptoms they wanted addressed, and/or what outcomes they hoped to achieve:

I think it's important for [PLWE] to understand what the goal of their treatment is … then they can align their treatment strategies with what the goal is. (Allied HP)

One highlighted that it took time and HPs’ guidance for PLWE to realise they had multiple goals:

All of [the PLWE clients] have at least 6 to 8 problems that they present with, and … [at the end of the consultation] they say, ‘OK, these are totally my goals, but I didn't know I had so many’. Part of it is that they have so many things on their mind … they get so overwhelmed that they don't even know where to start. (Specialist clinic manager)

Another clinician noted that for some PLWE, their goals precluded certain treatments:

Unfortunately, all the medical management things we have are contraceptives, essentially. So if your goal is to fall pregnant, then you're much more limited in what we can do to treat you. (Obstetrician-gynaecologist)

When working on goals, one HP stressed the importance of helping PLWE to take a longer-term view:

They have a working life, and their condition is getting in the way … so they choose a management option that maximises their ability to work. They don't think about that they won't always have such a high value on their working life. So their values can be changeable, but they don't realise it … they're not used to thinking about themselves in that way. (Complementary medicine practitioner)

One HP indicated that their clients wanted symptoms to be treated concurrently, rather than prioritising certain goals:

[My clients] feel like it minimises what's happening for them, that ‘actually, endo isn't just about one symptom, and I can't just pick one that I would want to treat well and have to put up with the rest’. (Allied HP)

### Sub-theme 1a: stepwise approach to treatment

Starting with the most conservative, least invasive treatment that might address symptoms (i.e. nonsteroidal painkillers) before trying hormonal medications or surgery was a popular protocol among HPs in this study. One noted this approach was perceived as being palatable to PLWE:

The simpler and cheaper and more available and less problematic the intervention is, the more likely you are to have people take it up. (GP)

Another compelling reason to try new treatments sequentially was to enable PLWE to pinpoint what was responsible for any change in symptoms:

Do one thing at a time to make sure that we know that if there's a change it's because of this, not because of the myriad possible things. (Complementary HP)

For one obstetrician-gynaecologist, three months of medical management coupled with allied and complementary health services was generally ‘a way to minimise the surgery that people are getting'. Unfortunately, this stepwise approach was sometimes at odds with patient expectations, as explained by this HP:

I think [PLWE] have an idea in their head that they put up with things for as long as they can and then when they can't, the next option is surgery … they feel in their head like ‘I’ve waited … I’ve tried all these different things, now I need surgery’. So they feel a little bit frustrated if that's the start of a very long journey for them to actually access surgery. (GP)

Several HPs reported that some PLWE were reluctant to consider treatment options other than surgery.

### Theme 2: HPs’ lack of time and perceived lack of knowledge

Every HP participant in this study specialised in endometriosis and its management, but many cited a lack of up-to-date knowledge about endometriosis as a concern across the general medical community:

Unfortunately, [PLWE] need to get a whole pile of education first so they can sort of almost interrogate the doctor. I’m somewhat ashamed to think that my profession is such that [PLWE] have to educate themselves before they come and see the doctors. (Obstetrician-gynaecologist)

HPs acknowledged this perceived lack of quality information from clinicians led to PLWE turning to social media communities for support and information, but reported that these communities could be a double-edged sword. On the positive side, reading about others’ experiences and connecting with fellow PLWE provided great support:

I think it normalises their symptoms, or it makes them feel like they’re not the only one. Facebook group forums … sometimes have more of an impact on what [PLWE] want than the people closest to them. (Obstetrician-gynaecologist)

However, concerns were raised about the sometimes negative skew of social media affecting people psychologically and physically:

There can be a negative spiral in some of those conversations … Anger doesn’t help pain, and pain is the main problem with endo. (Allied HP)

Furthermore, it was stated that popular overseas-based sites could mislead PLWE:

[Overseas website] doesn’t reflect the state of care that we receive in Australia or New Zealand … then we have a situation where people think that they’re getting sub-adequate care because they can’t access this [overseas] style of endometriosis surgery … to me it’s scaremongering. (Specialist practice nurse)

A lack of time in consultations was cited as a barrier to collaborative decision-making, with one GP criticising the public health system for financially rewarding shorter consultations over the longer appointments required for optimal SDM in a complex chronic condition like endometriosis:

[Doctors should not be] financially disadvantaged for having a long appointment … you need to take time [to treat endometriosis properly]. (Specialist clinic GP)

This problem was not limited to GPs, with one HP reporting:

Most specialists are very time-poor, their time limit is about 10-15 min … that would probably get you through them reading the notes and then making a decision for the [PLWE]. There's little time to actually explain all the benefits and the side effects. (Specialist clinic manager)

All HP participants in this study were very open to complementary and allied health therapies, however many described other doctors and specialists as being ignorant or closed off to potential treatments outside of their domain:

If your only solution is a hammer, then every problem becomes a nail. (Obstetrician-gynaecologist)

One HP clarified why a singular focus of some clinicians was problematic for decision-making:

[Some GPs and surgeons] can be quite negative to holistic diet and lifestyle measures, and I think that comes into the decision-making process for a person because they think, ‘well, that's not going to touch the sides if … the doctor's not recommending that’. (Complementary HP)

### Theme 3: patient-centred care and SDM

Every HP in this study emphasised the need for patient-centred care and SDM to facilitate endometriosis treatment decision-making, with one obstetrician-gynaecologist noting this started by simply listening:

I can't tell you how many times people come to me saying they’ve been ignored for years. So actually listen to what they’re saying. (Obstetrician-gynaecologist)

Unfortunately, all HPs recalled seeing PLWE who felt they had not been heard by prior clinicians, making them cautious about trusting new HPs and trying different treatments:

I think about 90% of [PLWE] … have a history of being reluctant with any HP because [previous clinicians they have seen] make the decision on their behalf … they felt like they … weren't part of that shared decision-making … and then they have complications that they weren't informed of. (Specialist clinic manager)

In extreme cases, HPs noted that PLWE felt they had been removed entirely from the decision-making process:

They come to me and feel like they haven't *had* a decision. They feel like they’ve been pushed down a certain pathway without other choices. (Complementary medicine practitioner)

Participants unanimously described the need to empower PLWE to play their role in SDM:

[PLWE have to be] in charge, because often when people are in pain they feel like they have lost autonomy and efficacy. So we need to put them back in the middle. (Specialist clinic GP)

To this end, participants talked about facilitating decision-making, rather than leading the process:

We try to support them in their decision by not trying to persuade them in any direction and trying to help them recognise what matters to them. (Complementary medicine HP)

I think [PLWE] are astute and they don't want to be told, rightly, what to do – they want to make their own decisions. And we need to facilitate that. (Obstetrician-gynaecologist)

### Sub-theme 3a: the effect of patient capacity on decision-making

All HPs reported that a key part of helping PLWE decide about treatments was recognising that resources (e.g. time, money, and physical and psychological capacity) were limited:

The less intervention, the less cost, the less effort, the more likely you are to get benefit from it, you’ll find probably more people will be willing to use it. [The use of painkillers] might be more common than physio and exercise programs, because [the latter] cost time and they cost money (Specialist clinic GP).

It was here that priorities played a key role:

It's a complex disease, so there's always going to be a list of things they maybe need to manage. But if, for example, the priority is to fall pregnant because they’ve been trying for three years, maybe their efforts need to be with the fertility doctors and not being at a physio. (Allied HP – physiotherapist)

The financial burden of treatments was reported to be hugely impactful on decision-making:

Cost is a very prohibitive barrier because it is a very expensive disease. (Allied HP).

Ongoing pain was also cited as significantly influencing PLWE's physical and mental capacity to engage with treatments:

Lifestyle stuff is obviously hard … Exercise is the cure of pain … but often people feel like they can't exercise because of their pain. (Specialist clinic GP)

One HP noted that it could be difficult to assess decisional readiness in PLWE:

I’ve been surprised by the amount of people who will see me, I’ll provide that advice, but they don't do it. You’d think if they’ve come to me they’re ready and they’re engaged. But the fact that they don't follow it through … you’re like, ‘OK, there were still barriers there’. (Specialist clinic nurse)

### Theme 4: patient decision-making blinded by hope

Setting realistic expectations was cited as a key factor in sound decision-making for PLWE, but this required HPs to have an up-to-date understanding of the condition. One HP remarked that a widespread, but outdated, view of endometriosis could lead to misguided hope:

We should be regarding this disease as a chronic, systemic inflammatory problem … If you were a doctor and you just assumed that endometriosis is a few cells outside the uterus and you tell that to the patient, then the patient would think, ‘OK, well, why don't you just remove those cells? That should fix me’. But it just doesn't necessarily work that way. (Obstetrician-gynaecologist)

Another participant noted that the overwhelming nature of endometriosis could lead to PLWE shutting down:

You think everybody would want treatment, but some people don't. I think what they’re really wanting is a magic wand for you to just take it away. (Specialist clinic GP)

More than half the HPs interviewed reported that the hope for a cure that did not exist sometimes clouded effective decision-making. Misguided hope could lead PLWE to treatments which had no evidential backing:

People go into a therapy with a high level of hope, which is why some people are willing to take on therapies which are not evidence-based … That indicates a certain level of need, or desperation. (Specialist clinic GP)

A complementary medicine therapist noted this desperation could lead PLWE to blindly accept a treatment suggestion from HPs without questioning whether it was really the right solution for their individual case, then feeling ‘quite a lot of shock' if it did not work as hoped. Symptom management was the main aim for HPs, but a few stated that nothing other than surgery was acceptable to some PLWE, who believed hormonal medications were ‘Band-Aid solutions'. Similarly, one HP stated:

You can get very, very good management strategies that will control that aspect of your symptoms [pain] … but the endo is still there. Some people are fixated on the fact that there's this thing inside them that needs to be gone. (Specialist clinic nurse)

Every HP discussed the need to be honest about exactly what they could − and could not − offer, yet several stated that the uncertainty of treatment outcomes left PLWE dissatisfied:

I can tell you broadly that [the pill can have side effects of] nausea and vomiting, gastric upset and weight gain and breast tenderness and yadda yadda yadda yadda. But what is it going to do for you? I don't know. And I think [PLWE] really wrestle with that. (Obstetrician-gynaecologist)

Not being able to offer guarantees about outcomes was noted by another participant:

I think there is a perception in our society that we can just fix everything all the time … We can help, but this isn't an all or nothing question … there might need to be a certain amount of accepting of symptoms and managing symptoms to the best possible capacity. (Specialist clinic GP)

### HP views on the potential value of a PtDA

HPs interviewed for this study were enthusiastic about the potential for a PtDA to empower PLWE:

It would help a lot to reduce their overwhelm, because you don't know what you don't know, so it’ll really help empower someone. And it probably will help the professional as well because it helps everyone in the end. (Complementary medicine HP)

A therapist noted that outlining the full range of treatments would be very helpful for those with doctors who did not have that information. One medical doctor said any knowledge that prompted discussion was a good thing:

I’m pro on people having lots of knowledge before coming in … using things online and coming to speak to me. (Obstetrician-gynaecologist)

One downside cited by a specialist clinic staffer was the lack of substantial evidence for many treatments in the endometriosis sphere, but a GP reflected the overall feeling of HP participants about a PtDA when they said:

Any structured approach is going to be useful and make sure that things are not missed, and if it helps [PLWE] … become more aware of what their needs or desires are, compared to what the possibilities might be, that might put them in a better position to ask the right questions. (Specialist clinic GP)

## Discussion

This is the first known study to explore HP perspectives of facilitators and barriers to decision-making of PLWE. Of the 13 HPs who were interviewed, 12 outlined *Identifying and Setting Priorities*as being a vital early step in decision-making, a view endorsed by researchers (Agarwal et al., 2019; Chapron et al., 2019; Royal Australian and New Zealand College of Obstetricians and Gynaecologists (RANZCOG), 2021). The alternative voice was an allied HP who reported their clients wanted endometriosis to be treated more holistically, taking all symptoms into account simultaneously. There is growing recognition of the need for multidisciplinary endometriosis care (Fang et al., 2024; Mechsner, 2023), with one qualitative study reporting that the lack of a holistic approach contributed to low satisfaction with symptom management in PLWE (Evans et al., 2022). However, the nature of endometriosis and its treatments necessitates a level of priority setting, depending on primary goals. For example, many hormonal treatments cannot be used by individuals with fertility-related concerns because these drugs are often contraceptives (Skorupskaite & Bhandari, 2021). Nevertheless, as understanding of the systemic nature of endometriosis develops (Taylor, 2019; Taylor et al., 2021), it is increasingly being recognised that PLWE need regimens that treat the condition as a whole rather than focusing on individual symptoms. This requires multidisciplinary teams that include allied health and complementary medicine practitioners alongside medical doctors such as GPs and obstetrician-gynaecologists (Agarwal et al., 2019; Fang et al., 2024).

Multi-disciplinary teams may be optimal, however accessing numerous therapies can be costly, and in *The Effect of Patient Capacity on Decision-Making* sub-theme, HPs’ views in this study reflected previous research documenting the considerable financial burden of holistic treatment for endometriosis (Armour et al., 2019a, 2019b). Under Australia's hybrid public-private health system, PLWE can theoretically access medical care free of charge, but the reality is that long waiting lists for publicly-funded specialists (Armour et al., 2022; Frayne et al., 2023) and very limited subsidies for non-medical therapies (Avila et al., 2020; Rosenberg et al., 2022) leave them waiting months or years for treatment, or force those who can afford it to pay hefty out-of-pocket expenses (Armour et al., 2019a, 2019b).

The *Capacity* theme did not focus solely on financial costs; HPs also reported the need for PLWE to have the physical and mental ability to engage with treatments. Decisional readiness has been extensively researched in other areas of healthcare (Pablos et al., 2020; Sherman et al., 2016; Witteman et al., 2021a, 2021b), but has not been examined in endometriosis. There are four subdomains of decisional readiness: worry, knowledge, uncertainty about the decision, and decision-making preparation (Witteman et al., 2021a, 2021b). PLWE have been found to experience a high degree of worry (Ruszała et al., 2022; Zarbo et al., 2022), report feeling uninformed about the condition and its treatments (Sherman et al., 2022), and experience high levels of uncertainty about potential treatment outcomes (Culley et al., 2013; Handelsman et al., 2023), suggesting decisional readiness is likely to be low in these individuals. Future research should explore decisional readiness in PLWE, and develop interventions that could boost this key component of SDM (Fisher et al., 2018; Keij et al., 2021). PtDAs have been shown to improve patient readiness to make a healthcare decision (Joseph-Williams et al., 2021).

HPs in this study also affirmed previous research outlining the complexity of understanding and treating endometriosis (van der Zanden & Nap, 2016; Zale et al., 2020), and pointed to a basic systemic barrier to providing optimal care. *Patient-Centred Care and SDM* were unanimously endorsed by all HP participants, but a few noted that the Australian public health system's predilection for short consultations served as a financial disincentive for doctors to take the time required to fully address PLWE's needs, reflecting prior research (Evans et al., 2022).

A further barrier to SDM was identified in the *Stepwise Approach to Treatment* sub-theme. HPs endorsed starting with the most conservative treatments and moving systematically through options before trying more invasive treatments. While this approach is endorsed by researchers and medical professionals worldwide (Mechsner, 2023; Vercellini et al., 2018), several HPs observed that PLWE new to them were sometimes fixated on surgery being the only option. The need for PLWE to self-educate (Holowka, 2022; Whelan, 2007) was reported, and lamented, by the HPs in this study, but it is vital that PLWE base their treatment decisions on the most up-to-date, valid information available (Omtvedt et al., 2022; Rowe et al., 2021). Latest endometriosis-related research recommends a move away from multiple surgeries because the operations carry inherent risks, such as adhesions, and bladder and bowel dysfunction (Armour et al., 2022; Saraswat et al., 2018), and the lesions frequently re-occur (Nirgianakis et al., 2021). However, a specialist clinic nurse said some popular overseas social media sites could be guilty of overstating the need for surgery, encouraging some Australian PLWE to dismiss consideration of other efficacious treatments.

Potentially misleading information aside, all HPs in this study touted the benefits of social media communities, mainly in giving PLWE support they lacked, as has been found previously (Holowka, 2022). However, one allied HP cautioned that the level of anger and negativity in some social media conversations could be problematic, pointing to the documented association between negative emotionality and increased pain (Yarns et al., 2022).

Another barrier to collaborative SDM cited by HPs in this study was what they perceived as a lack of education about endometriosis among the wider medical community, as described in the *HPs’ Lack of Time and Perceived Lack of Knowledge* theme. This has been raised in prior research, by both HPs (Grundström et al., 2016; van der Zanden & Nap, 2016) and PLWE (Navarria-Forney et al., 2020; Young et al., 2015), and has been reported as a key factor in eroding PLWE's trust in endometriosis healthcare providers (Mikesell & Bontempo, 2023). A good therapeutic alliance between HP and patient is essential for SDM (Santana et al., 2018), so it is unlikely PLWE who are sceptical about clinician expertise are engaging in a collaborative approach to decision-making. A decision support tool containing information reflecting the most up-to-date scientific knowledge could potentially improve HP's understanding of endometriosis and reset patient expectations, reducing conflict between the two and leading to more effective collaboration. However, a few HPs in this study observed that some PLWE would not accept anything other than surgery, even if their symptoms were managed by other treatments. The perceived need by some PLWE to be rid of ‘this thing inside them’ suggests a psychological barrier to optimal decision-making that warrants further investigation.

One striking finding from these data was the need for GPs and medical specialists to understand the potential of allied health and complementary medicine treatments in endometriosis symptom management. This sample included five practitioners from non-medical therapies and eight from medical practices, and all stressed the need for integrative care combining a range of medical, surgical and non-medical treatments for endometriosis. However, previous research shows that medical professionals are unwilling to recommend treatments outside of their specific expertise (Evans et al., 2022), and the HPs in this study confirmed this was often the case. While clinical trials are required to validate their effectiveness, initial studies indicate potential endometriosis symptom management using cannabis products (Carrubba et al., 2021), improving sleep (Li et al., 2022), physical exercise (Evans et al., 2019; Gonçalves et al., 2016), diet modification (Kumar et al., 2023), pelvic physiotherapy (Muñoz-Gómez et al., 2023; Wójcik et al., 2022) and mindfulness (Hansen et al., 2023; Moreira et al., 2023). It has been reported that 75% of Australian PLWE use self-management strategies such as these (Armour et al., 2019a, 2019b), but very few are confident about their knowledge of such treatments (Adamietz et al., 2021); further underscoring the need for a decision support tool that could boost knowledge in PLWE and HPs of the full range of valid treatment options.

The final theme identified in these data was *Patient Decision-Making Blinded by Hope*, and this is a subject that has been under-researched in endometriosis. Positive emotions such as hope have been found to favourably affect people's intention to change their health behaviour (Nabi & Myrick, 2019), including in endometriosis screening (Worsdale & Liu, 2023). Research reports that optimal levels of hope, defined as the belief that personal goals can be achieved (Snyder et al., 1991), are associated with improved outcomes when people are sick (Musschenga, 2019), and help them adapt to chronic illness (de Andrade Alvarenga et al., 2021; Yücens et al., 2019). However, aiming for impractical goals that do not reflect realistic limitations can lead to worse psychological outcomes, as well as being financially costly as people try treatments unlikely to ease their symptoms (Eijkholt, 2020). PLWE frequently report feeling hopeless (Calvi et al., 2024; Cox et al., 2003; Whelan, 2007). However, many HPs in the current study discussed how misguided hope could interfere with sound decision-making. A specialist clinic GP pointed out that, despite all attempts at treatment, the unfortunate truth was that some PLWE would have to set realistic expectations, and simply manage their symptoms to the highest degree possible. Prior research has found that acceptance of their illness is associated with higher quality of life in PLWE (Bień et al., 2020; Márki et al., 2022).

Taken together, the themes identified from these qualitative analyses indicate that HPs believe individuals with endometriosis are not receiving the support they require when choosing treatments for symptom management. The complexity of the condition and the difficulty in finding an individualised treatment plan were acknowledged by all HPs, who advocated for patient-centred care, actively listening, and more time in consultations. The majority described trying to clarify goals to identify what was important for each individual, and recounted efforts they made to ensure PLWE had the required knowledge to make an informed decision. However, they were less than complimentary about the wider medical community, pointing to other clinicians’ perceived lack of attentiveness and lack of knowledge, including an inability or unwillingness to recommend allied health and complementary therapies. However, there are two sides to SDM, and while no HP said anything negative about PLWE, they did allude to issues that might hinder effective decision-making. The first factor was some PLWE's insistence on surgery, despite other potentially efficacious treatments being available. Related to this was PLWE's tendency to access information via social media communities, which provided them with a source of support but could potentially misinform. Lastly, PLWE's hope for a cure that did not exist was described as a factor that potentially obfuscated quality decision-making. These data confirmed that many PLWE do not currently have adequate resources and support to make complex decisions about their treatment, and the HPs welcomed the idea of a PtDA to help fill that gap, noting that it could be also used by clinicians.

This study has for the first time recorded HPs’ insights into the decision-making processes of PLWE, but some limitations must be acknowledged. Efforts were made to canvas a wide range of HP views, but not every area offering treatment to PLWE was represented (Becker et al., 2022; Kalaitzopoulos et al., 2021). In addition, the selection of potential participants was not random: firstly, invitations were sent to HPs known to be active in the endometriosis sphere; and secondly, only 20% of invited HPs volunteered and participated, which may have resulted in recruitment and volunteer biases potentially skewing results. Additionally, it is crucial to acknowledge the potential for researcher bias to affect data collection and/or analysis in qualitative research, therefore reflexivity statements from LF, CP and KS are provided in Supplementary Materials C. Lastly, the research team created and ran this study predicting that the endometriosis community would most probably benefit from a PtDA, and this could have inadvertently coloured data collection and interpretation.

The results of this study indicate a support tool such as a PtDA could significantly assist PLWE in treatment decision-making. The HPs in this study were enthusiastic about the potential utility of a PtDA, with some noting it could also be a good tool to guide less knowledgeable clinicians, and others predicting that it could streamline consultations by giving PLWE the knowledge to ask salient questions of their healthcare team. The results of this study will help to inform the development of a co-designed, consumer- and HP-informed PtDA to support and facilitate the treatment and management decision-making process for PLWE. This interactive online tool, based on global best-practice guidelines (Elwyn et al., 2006), will include easy-to-read information on the full range of evidence-based endometriosis treatments and guidance to help users decide what is the best choice for them (Geukens et al., 2018; Metzemaekers et al., 2021), with the aim of empowering PLWE with the knowledge and confidence to play their part in SDM.

## Author contribution

All authors agree to be accountable for all aspects of the work, have approved the contents of this paper, and have agreed to the submission policies of the Health Psychology and Behavioral Medicine. Individual contributions of each author are as follows:

- Lynda Fallon: conception and design; data collection; analysis and interpretation of the data; drafting the paper; revising it critically for intellectual content; and final approval of the version to be published.
- Kerry A. Sherman: conception and design; analysis and interpretation of the data; drafting the paper; revising it critically for intellectual content; and final approval of the version to be published.
- Annie Y. S. Lau: conception and design; revising the paper critically for intellectual content; and final approval of the version to be published.
- Donna Ciccia (endometriosis consumer investigator): conception and design; revising the paper critically for intellectual content; and final approval of the version to be published.
- Tanya Jane Duckworth (endometriosis consumer investigator): conception and design; revising the paper critically for intellectual content; and final approval of the version to be published.
- Chantelle Pereira: analysis and interpretation of the data; revising the paper critically for intellectual content; and final approval of the version to be published.
- Emily Kopp: data curation and final approval of drafts.
- Valentina Perica: data curation and final approval of drafts.

## Supplementary Material

Supplementary Materials.docx

## Data Availability

Due to the sensitivity of the data, it is not publicly available.
