# Supplementary material for: Perceived challenges in treatment decision-making for endometriosis: healthcare professional perspectives
Source: Health Psychol Behav Med. 2024 Aug 1;12(1):2383469. doi: 10.1080/21642850.2024.2383469 (PMC11295683; doi:10.1080/21642850.2024.2383469)
Supplement: Supplementary Materials.docx [file RHPB_A_2383469_SM1955.docx]

**Supplementary Materials A: Questions for Endometriosis Healthcare Professionals**

Thank you so much for your time today, I really appreciate you sharing your knowledge and experience with me for our study, which aims to explore the challenges patients face when making decisions about managing their endometriosis. Are you happy for me to record this sessions?

1. What do YOU feel are the key decisions patients need to make about their endometriosis management?
2. Which aspects to do you feel patients wrestle with most when making those decisions?
3. Which aspects do you think they find easiest when making those decisions?
4. How can healthcare professionals help with a patient’s decision-making process?
5. Do you think anyone else can play a valuable role? (Prompt: family, friends, support groups)

Patient decision aids have been found to help individuals make an active and informed choice that best suits their values and minimises any psychological conflict they may feel about those choices. They do this by presenting reliable, evidence-based information about available options for medical treatments and disease management, as well as conducting a values clarification exercise. PDAs have been found to help in range of different health decisions about treatment or screening options ...

1. Do you think a web-based patient decision aid could be helpful for people with endometriosis?

- How do you think it would help?
- Can you see any disadvantages in a patient using a decision aid?

Thank you for your time today, it’s greatly appreciated. Please let me know if you’d like me to send you the results of this research, and please don’t hesitate to get in touch if you have anything further to add.

**Supplementary Materials B: Additional Quotes**

**Identifying and Setting Priorities – Additional Quotes**

- [Patients] need to prioritise, have a think about their hierarchy of needs and requirements at that point in time because that changes quite a bit for people - one of the obvious things obviously is when people are considering trying for a pregnancy or trying to avoid a pregnancy. (Specialist clinic GP)
- I'm really focused on like goal-orientated treatment because the different treatments will depend on the goals ... Sometimes they've got multiple goals, sometimes they have pain and bowel [conditions] and fertility [issues], and that actually makes it pretty difficult. (Obstetrician-gynaecologist)
- [Patients need to ask] ‘Who do I have around me to support me?’. And I suppose the precursor to that is, ‘who do I need on my team? Who's actually really important for me?’. Because, obviously, endo presents very differently and everyone's priorities are very different as well. (Allied health practitioner)

**HPs’ Lack of Time and Perceived Lack of Knowledge**

- They really do need a lot of time. They have a range of problems and those are by-products from their pelvic pain. So it's a lot to address and most GPs get so overwhelmed. (Specialist clinic manager)
- Unfortunately, I think the patients really need to have a huge amount of education ... That's unusual in medicine because, you know, you usually go along to your doctor and say ‘these are things that's happening to me, you tell me what might be wrong and what can I do about it’. (Obstetrician-gynaecologist)
- I’m just wondering whether they're given enough time to ask questions, you know, to take information away, whether that's written material, whether that's an understanding of what surgery can and can’t do, what medication can and can't do ... I think there's a difference between consent and informed consent. (Allied HP)
- Part of our role as a GP, I think, we're like a concierge, so I try really hard to match the patient to the specialist ... And just matching personalities. Some people like a bit of direction, but some people like warm and fuzzy. So I feel that’s part of my job [matching the patient with the right specialist] because if they don't get on with the specialists, the specialists could be telling them the right thing, but they won't do it. So it's really important that their personalities match. But that's something you can only judge with time and experience. (Specialist clinic GP)
- A lot of the distress that [my clients are] feeling, I think if there had been a discussion at the outset that the laparoscopy isn't going to address [everything] it would help with that expectation, or the hormonal medication won't address that, but it will address this. The adjustment process would be smoother. (Allied HP)

**Patient-Centred Care and SDM**

- I highlight that this is a subjective disease that this is a quality-of-life thing. And nobody can tell me whether their quality of life is better or worse than the patients themselves. (Obstetrician-gynaecologist)
- “I tell them all the pros and cons, and make it clear it is their choice whether they use it and how much they use. There's not one fix. There's a whole lot of things that need to be done. The way I practice is that people can choose from that menu. I would never tell somebody they had to do one thing or the other. (Specialist clinic GP)
- [Care] needs to take that person-centred approach. Regardless of your professional domain that you are coming from, it's about spending the time listening to what are the main impacts for the person. (Allied health professional)
- The idea is to try to listen to the [patient] and not overstate the benefits for any particular approach so that they're making a decision based on realistic expectations. I think that it's inhumane to confuse them or encourage them to make a sort of practitioner- or a modality-centred kind of choice rather than a person-centred choice. (Complementary medicine practitioner)
- Saying ‘these are the treatments that you have available that we know are backed by science, and you are in the centre of all these options. And you are always the one to make the decision of what suits your life and circumstances. We provide the treatments, you decide what suits you’. But I think them knowing their treatment options is one of the barriers. (Allied HP)
- Because obviously a health professional is one piece, we need a few people to make that puzzle, with the person in the middle. Connecting everyone together. (Allied HP)

**The Effect of Patient Capacity on Decision-Making**

- The ability to judge the expertise of the person who is offering their services is a tricky one as well. (Specialist clinic GP)
- It's really expensive, in particular, if you're trying to do four things at once – naturopath, physio, specialist, the medications. I think because we have an hour with people, we hear a lot about the burden of disease that potentially the specialists and the consultants aren't hearing because they've got 15 minutes. (Allied HP)
- In terms of conflict, I think it's more often around this fear that they're going to make the wrong decision. (Allied HP)
- [Patients] need to actually advocate - and that's a big decision in itself, I think, because it takes actually a lot of guts to advocate for yourself. (Allied HP)

**Patient Decision-Making Blinded by Hope**

- [Patients] come into the clinic and they think they've made a decision, but they've made it based on an unrealistic expectation. (Complementary medicine practitioner)
- What made the [endometriosis] community wild was when they found out about the guidelines don't recommend excision over ablation. That is because we don't have the evidence to say that excision is better, it's very difficult to study ... Evidence that we have for pain outcomes is almost conclusively damning that excision surgery does not improve long-term pain. And the community was so upset about what the guidelines said, but you have to remember that the guidelines can only say what the evidence says. And the evidence is very poor across all domains. Hormonal, physical management, alternative therapies. (Specialist clinic nurse)
- These things take time. It's taken years to this point. So we're not going to, there's no magic bullet here. It's not going to happen overnight, but we can improve it. (Allied HP)
- I guess the final thing that I always tell them is that surgery doesn't cure it. So you know we could operate on you and get rid of your disease, but potentially in a year or two, you could be back with the same pain. So being realistic about what the goal outcomes are as well is important. (Obstetrician-gynaecologis)
- I think this is an incurable disease that we have to manage symptomatically, symptom management. (Specialist clinic nurse)

**Need for a PtDA**

- I wish I had that when I was diagnosed. And it would help a lot to reduce their overwhelm, because you don't know what you don't know, so it'll really help empower someone and help. (Allied HP)
- It probably will help the professional to be honest as well because it helps everyone in the end. (Allied HP)
- I think a decision tool linked with a healthcare team that's inviting the time and the accurate information, I think would be really powerful. (Allied HP)

**Supplementary Materials C: Researcher Reflexivity Statements**

**Reflexivity Statement: Lynda Fallon**

In qualitative research it is imperative to engage in a reflective process to acknowledge how my personal experiences, biases and beliefs may have affected how I carried out data collection and analysis for this project.

During the data collection stage for this project, I was also butting heads with numerous HPs trying to find a cause of extreme neck pain for my 14-year-old daughter. The conclusion was the diagnosis of a potentially life-threatening condition, after at least nine HPs dismissed our concerns. There is no doubt that I felt antipathy towards HPs in general at that time, so during each interview for this study I made a conscious effort not to let it affect my interactions with them, however I am not in a position to judge how successful I was. This general dissatisfaction with the health system was counter-balanced with feeling very grateful to these HPs, who were freely giving their valuable time and insights, compared to many who had declined my request. In the end, I came away from those interviews impressed by all the HPs’ skill sets and care for endometriosis patients, and I think I have been transparent about that in the paper.

This study was conducted alongside a second study (Perceived Challenges in Treatment Decision-Making for Endometriosis: People Living With Endometriosis Perspectives), which collected data from focus groups of people with endometriosis. The participants in those focus groups were highly critical of the health system and HPs, but most of the HP interviews (10 of the 13) were held before those focus groups, so I do not believe the patient experiences affected how I gathered or analysed the HP data. In addition, each HP was asked set questions which were formulated by Authors 1 and 2 before any interview or focus group was held.

While not having endometriosis myself means I do not have an understanding of what it is like to live with this condition, I believe this was a bonus when talking to HPs, because I have not experienced the commonly cited issue of having endometriosis symptoms invalidated. However, as a young girl I was told by doctors to “get on with it” when I had debilitating period pain, and, while I don’t think I harbour any resentment over that, it should be acknowledged as a potential bias on my part. In addition, in conducting the literature review for this research, many papers discussed endometriosis patients’ dissatisfaction with clinicians, which may have coloured my view.

Despite my best efforts to remain impartial, I acknowledge that this paper reflects the interplay between my personal identity as a middle-aged, educated and middle class woman and my academic commitment to producing valuable, unbiased knowledge. I have tried to ensure this research is transparent and rigorous, while faithfully reproducing the views of HPs.

**Reflexivity Statement: Kerry A. Sherman**

I acknowledge that my involvement, interpretation and analysis of the data collected in this qualitative research is a reflection of my world view and past experiences. Specifically, I am an experienced, female researcher and university academic. I have extensive experience working in different aspects of health psychology including breast cancer, head and neck cancer, prostate cancer, and endometriosis. My work in these areas has consistently entailed close interactions with consumers and consumer-based organisations. My work is motivated by the desire to improve the psychological wellbeing of individuals living with chronic conditions, such as endometriosis, and to develop interventions to provide psychological support in decision making, and in managing symptoms such as body image concerns and psychological distress.

Added to this experience as a researcher, I am also an individual with lived experience of endometriosis. My interest and motivation in this specific research focused on decision-making in endometriosis is therefore a reflection of both professional and personal reasons.

**Reflexivity Statement: Chantelle Pereira**

I am a graduate interested in pursuing a career in endometriosis research. I was keen to assist with this study to get a better understanding of qualitative research and the views of medical professionals working with those diagnosed with endometriosis.

Having lived experiences of endometriosis provided a broader context and motivation to be involved with this research. The transcription and initial coding stages of the project required discipline and consideration on my part. I acknowledge that my interpretation of transcripts was undertaken within my personal understanding of this condition. Having a history of negative experiences with healthcare professionals in the past, I tried my best to keep an open mind with respect to how I interpreted the data to ensure this did not bias my judgment.

I was encouraged by my colleagues to be confident in what I felt was important and meaningful in the dataset. This required me to take ownership of my work but also to keep in mind that I am just one researcher as part of a team and that collaboration was important to maintain a good sense of the goals of our analysis.
